# In silico approaches for the identification of potential allergens among hypothetical proteins from *Alternaria alternata* and its functional annotation

**DOI:** 10.1038/s41598-024-55463-1

**Published:** 2024-03-20

**Authors:** Rajamanikandan Sundararaj, Amala Mathimaran, Dhamodharan Prabhu, Balajee Ramachandran, Jeyakanthan Jeyaraman, Saravanan Muthupandian, Tsehaye Asmelash

**Affiliations:** 1 Department of Biochemistry, Centre for Drug Discovery, Karpagam Academy of Higher Education, Coimbatore, 641021 India; 2 Structural Biology and Biocomputing Lab, Department of Bioinformatics, Alagappa University, Karaikudi, Tamil Nadu 630 004 India; 3 Department of Biotechnology, Centre for Drug Discovery, Karpagam Academy of Higher Education, Coimbatore, 641021 India; 4 Department of Pharmacology, Physiology & Biophysics, Chobanian & Avedisian School of Medicine, Boston University, 700 Albany Street, Boston, MA 02118 USA; 5 Department of Pharmacology, Saveetha Dental College, Saveetha Institute of Medical and Technical Sciences (SIMATS), Chennai, 600077 India; 6 Department of Medical Microbiology and Immunology, College of Health Sciences, Mekelle University, Mekelle, Tigray Ethiopia

**Keywords:** *Alternaria alternata*, AlgPred, SDAP, Structure–function relationship and drug discovery, Infection, Biochemistry, Biotechnology, Computational biology and bioinformatics, Drug discovery, Ecology, Microbiology, Environmental sciences, Health care

## Abstract

Direct exposure to the fungal species *Alternaria alternata* is a major risk factor for the development of asthma, allergic rhinitis, and inflammation. As of November 23rd 2020, the NCBI protein database showed 11,227 proteins from *A. alternata* genome as hypothetical proteins (HPs). Allergens are the main causative of several life-threatening diseases, especially in fungal infections. Therefore, the main aim of the study is to identify the potentially allergenic inducible proteins from the HPs in *A. alternata* and their associated functional assignment for the complete understanding of the complex biological systems at the molecular level. AlgPred and Structural Database of Allergenic Proteins (SDAP) were used for the prediction of potential allergens from the HPs of *A. alternata*. While analyzing the proteome data, 29 potential allergens were predicted by AlgPred and further screening in SDAP confirmed the allergic response of 10 proteins. Extensive bioinformatics tools including protein family classification, sequence-function relationship, protein motif discovery, pathway interactions, and intrinsic features from the amino acid sequence were used to successfully predict the probable functions of the 10 HPs. The functions of the HPs are characterized as chitin-binding, ribosomal protein P1, thaumatin, glycosyl hydrolase, and NOB1 proteins. The subcellular localization and signal peptide prediction of these 10 proteins has further provided additional information on localization and function. The allergens prediction and functional annotation of the 10 proteins may facilitate a better understanding of the allergenic mechanism of *A. alternata* in asthma and other diseases. The functional domain level insights and predicted structural features of the allergenic proteins help to understand the pathogenesis and host immune tolerance. The outcomes of the study would aid in the development of specific drugs to combat *A. alternata* infections.

## Introduction

Airborne fungi and their spores are the most common bio-aerosols that are inhaled by humans. It causes numerous health issues to human notably allergic rhinitis and asthma^1^. *Alternaria alternata* is a ubiquitous saprophytic, air-borne fungus found commonly in the environment. *A. alternata* plays a major role in the asthma development of sensitized individuals^2^ and another study reported approximately, 80% of asthmatic patients are sensitized to *A. alternata*^3^. Several life-threatening exacerbations of asthma have also been strongly linked to *A. alternata* exposure^4^. Furthermore, *A. alternata* is strongly associated with the development of Type I hypersensitivity, triggering an IgE response against allergens and leading to the release of pharmacological mediators, such as histamine-IgE-sensitized mast cells, resulting in acute inflammatory reactions like asthma or rhinitis. The Global Asthma and Allergy European Network and the National Health & Nutrition Examination Survey have reported the occurrence of allergic rhinitis in the United States and around Europe by 12.9% and 8.9%, respectively^5^.

Alternaria spores are the most common fungal allergens, often found in areas with humid climates and also in arid regions^4^. Higher concentrations of spore counts are assessed in outdoor environments, but the spores may also enter the indoor environment through an infiltration process or ventilation, apart they can also be carried by indoor occupants^6^. Because of their larger spore size (23–34 µm × 7–10 µm), it is frequently found in indoor dust particles and cannot be eliminated through normal filtration methods. Though the *Alternaria* spores are more common in an outdoor environment, the indoor environment has also been the secondary source of exposure for the colonization of invaded spores in the building materials and indoor space^7^. Until now, 17 potential allergens of *A. alternata* have been reported on the International Union of Immunological Societies (IUIS) website and the Allergome database^8^. However, Alt a 1 is considered a major allergen among 17 allergens of *A. alternata* and it was predominantly found in more than 85% of *Alternaria*-induced allergic patients^9^.

Allergens are a class of proteins that can elicit the powerful T helper lymphocyte type 2 (Th_2_) responses, culminating in excessive IgE antibody production for the development of allergic diseases. Antigen recognition and uptake by innate immune cells is the primary defense step against allergic reactions and produces excessive IgE antibodies, sensitizing and triggering mast cells^10^. Type I hypersensitivity reactions are classified into immediate and late-phase reactions. The immediate reaction occurs within a few minutes (5–30 min) and subsides in 60 min, resulting in Thr release of inflammatory mediators. The late phase reactions occurs in 3 to 4 h (starting in 2–8 h and lasting 2–3 days) and is cell-driven process leading to cellular infiltration and mediator release^11^.

Asthma and allergic inflammations result from dysregulated Th_2_-like airway inflammatory responses to the environment^12^. These responses are mediated by CD4^+^ T cells polarized towards Th_2_ cell phenotype and help B cells for IgE expression^13^. Th_2_ also interacts with other cells such as eosinophils through IL-5; smooth muscle cells through IL-9; epithelial cells and keratinocytes through IL-13 and epithelial cells through IL-31, that drive the pathogenic characteristics of asthma, such as increased IgE, airway hyperresponsiveness, excessive mucus production, airway remodeling, and airway eosinophilia^14^. The interaction between cytokines IL-5, IL-13, and their receptors on the B-cell surface is the first signal for antibody class switching. The second signal involves an interaction between CD40 on B cell and CD40L on T cell leading to the production of IgE antibodies. The transcriptional factors such as BSAP (B cell specific activator protein), NF-Kβ (nuclear factor kappa B), and STAT6 (singal transducer and activator of transcriptorion) are identified to bind in the promoter sequence of various allergic reaction-associated genes^15^. Comparatively upregulation of mRNAs specific to the chemokine proteins (eotaxin, MIP-1α, MIP-2) and chemokine receptors (CCR-1, CCR-2, and CCR-5) were observed in the *A. alternata* spores infected lung allergy patients^16^. Expression of CCR-3 in lungs and Th_2_ cytokine (IL-4, IL-5, and IL-13) secretion in the BAL (bronchoalveolar lavage) was also additionally observed.

*A. alternata* is a major fungal pathogen exhibiting harmful effects on human and plant health. In the present study, we have identified the potential allergens from the hypothetical proteins (HPs) of *A. alternata*. Sequence and structure-based computational approaches were used to characterize the functions of the HPs. Decoding the complete proteome aids in identification of potential virulence causatives and allergenic proteins of the pathogen and thereby pave the way for drug and vaccine discovery. Numerous literature reports states the use of bioinformatics-based approaches for the design and development of drug and highly effective vaccines to treat infections caused by the bacterial pathogens^17–22^.

## Materials and methods

### Sequence retrieval and dataset analysis

The complete HP sequences of *A. alternata* were retrieved from the NCBI database using their primary accession numbers in FASTA format. The sequences of all 11,227 HPs were subjected to the computational prediction for the identification of potential allergens. Furthermore, the identified allergens are functionally annotated using a well-optimized series of bioinformatics tools.

### Allergen prediction: AlgPred and SDAP

AlgPred, a web-based (http://www.imtech.res.in/raghava/algpred) allergen prediction tool was used to predict the possible allergenic proteins present among 11,227 HPs of *A. alternata*^23^. AlgPred also predicts the potential IgE epitopes in the subjected 11,227 HPs. The tool uses different approaches for the prediction of allergenic proteins, which includes motif based techniques, machine learning and hybrid approach^24^. The protein predicted to be an allergen by most of the approaches has a high probability to be an allergenic protein. The possible allergenic proteins predicted in the AlgPred tool are subjected to the Structural Database of Allergic Proteins (SDAP), to investiage the cross-reactivity between known allergens (http://fermi.utmb.edu/sdap/)^25^. In order to determine the distantly related sequences, the physical–chemical amino acid descriptors *E1–E5* were used to locate sequences with similar chemical properties. In *E1–E5* descriptors, the similarities between the two sequences are examined with the property distance function *PD*. Each amino acid is represented as a vector and these vectors are generated by the method of metric multidimensional scaling of 237 physical–chemical properties for the naturally occurring 20 amino acids.

### Physicochemical properties and sub-cellular localization

ProtParam tool in the Expasy server (http://web.expasy.org/protparam/) and PDB Goodies were employed to compute the physicochemical properties of HPs^26,27^. Theoretical calculation of various physicochemical properties such as molecular weight, aliphatic index, isoelectric point, instability index, extinction coefficient, and grand average of hydropathicity (GRAVY) was calculated for the selected 10 HPs (Table 1). The Wolf PSORT^28^ and CELLO^29^ servers were used to predict the subcellular localization of the potential allergens**.** Wolf PSORT converts given sequences into numerical localization features based on sorting signals, amino acid composition, and functional motifs. Upon conversion, the simple k-nearest neighbor classifier is used for the protein subcellular location prediction. CELLO uses a two-level SVM (Support Vector Machine) classifier and homology search method to annotate the sub-cellular localization of HPs (Table 2).

**Table 1:** Physico-chemical properties predicted for the potential allergens of *A. alternata*.

S. no	Protein A.C	Mw	pI	− charged amino acid	+ charged amino acid	Extinction co-efficient	Instability index	Aliphatic index	GRAVY
1.	A0A177D895	21,501.08	7.95	13	16	25,450	25.84	38.58	− 0.289
2.	A0A177DEP8	14,981.04	6.29	16	15	13,980	47.78	92.47	0.101
3.	A0A4Q4NGZ8	12,345.50	4.06	25	10	6990	25.23	84.49	− 0.342
4.	A0A4Q4NJR8	45,038.53	5.75	42	37	58,870	41.43	61.67	− 0.453
5.	A0A4Q4N975	65,087.68	4.77	77	46	59,250	59.68	60.58	− 0.403
6.	A0A177DU49	67,982.11	5.04	103	74	43,430	56.26	68.91	− 0.921
7.	A0A4Q4N5B7	11,239.49	4.09	23	9	6990	54.04	87.39	− 0.086
8.	A0A4Q4NRZ2	68,010.17	5.04	103	74	43,430	56.26	69.22	− 0.917
9.	A0A177DB16	50,751.33	7.54	28	30	52,435	50.50	59.82	− 0.108
10.	A0A4Q4NI20	51,485.77	6.18	30	29	44,235	46.10	54.46	− 0.204

**Table 2:** Sub-cellular localization annotation of hypothetical proteins of *A. alternata.*

S. no	Protein A.C	Wolf PSORT	Cello
1.	A0A177D895	Extracellular	Extracellular
2.	A0A177DEP8	Cytoplasmic	Chloroplast
3.	A0A4Q4NGZ8	Cytoplasmic	Cytoplasmic
4.	A0A4Q4NJR8	Extracellular	Extracellular
5.	A0A4Q4N975	Mitochondria	Extracellular
6.	A0A177DU49	Nuclear	Nuclear
7.	A0A4Q4N5B7	Cytoplasmic	Cytoplasmic
8.	A0A4Q4NRZ2	Nuclear	Nuclear
9.	A0A177DB16	Extracellular	Extracellular
10.	A0A4Q4NI20	Extracellular	Nuclear

### Sequence-based functional annotation

The identified allergens in *A. alternata* were extensively analyzed using CDD (Conserved Domain Database), InterPro, and Pfam to characterize the functional domains by utilizing the sequences of these HPs^30–32^. CDD inspects the functional characteristics of protein sequences by using the heuristics BLAST algorithm, and searches against a complete collection of domains to identify the structural and functional domains in the protein sequences^33^. InterPro scan combines multiple resources for motif discovery which predicts the information of protein domains, families, and functional sites. Protein sequence motifs are the signatures of protein families that are often used in predicting the function of the protein, especially in the case of metabolic enzymes; these motifs are associated with catalytic functions. Pfam is defined by multiple alignments and profile hidden Markov model (HMM) to define the family-representative sequences. Pfam uses the HMM algorithm to search the target sequence against the UniProt Knowledgebase (UniProtKB) to predict family relationships^34^.

### Structure modelling and validation

The protein structural folds are highly conserved than sequences. Thus, structure-based functional annotation of the HPs are considered more reliable than sequence-based function assignment. The three-dimensional structure of predicted allergens was determined using the Phyre2 (comparative homology)^35^ and Robetta (de novo)^36^. In the absence of the structural homology in repositories, Robetta builds the three-dimensional structure of the targeted allergic proteins by the de novo fragment insertion method. The Monte Carlo local structure search algorithm was used for energy minimization and optimization. Both knowledge-based and physically-derived scoring terms were used to score the quality of the generated models. PROCHECK program was used to validate the reliability of the generated structures by analyzing the overall structure and residue-by-residue geometry of proteins^37^. ProQ, a neural network method was also used to predict the quality of the predicted structures^38^. Models showing a high LG score and MaxSub score were selected for function prediction studies.

### Structure-based functional prediction

The predicted structures of the HPs are then used as similarity search queries in ProFunc and DALI servers for the structure-based function prediction^39,40^. ProFunc uses secondary structure elements (SSEs), SURFNET algorithm, residue conservation, and nest analysis on query structure to identify similar functional motifs or close associations to the experimentally annotated proteins. DALI uses a weighted sum of similarities of intra-molecular distances to classify the structurally similar proteins in the PDB databases related to our input structure. The list of structural neighbors is sorted by pairwise structural similarity score (Z-score). A higher Z-score implies the structures agree more closely in architectural details.

## Results and discussion

Advancements in the field of computational biology have developed several models namely the Hidden Markov Model (HMM), Neural Network (NN) model, and Support Vector Machine (SVM) to decode the biological phenomenon at the system level. The models and their associated methods are more efficient and accurate in annotating the functional properties of the proteins. We have used above-described models and methods to identify the potential allergens from the HPs of *A. alternata.* Further, the functions of the selected proteins were annotated based on their sequence and structural information. A total of 11,227 HPs of *A. alternata* were retrieved from the NCBI database and evaluated for their allergenicity using bioinformatics approaches.

### Allergenic prediction

The predictions of allergenic proteins through computational approaches are an important phenomenon in the development of an effective vaccine and therapeutics in pharmaceutical industries. FAO/WHO (FAO: Food and Agriculture Organization of the United Nations; WHO: World Health Organization), Codex Alimentarius Commission guidelines (2003) have recommended various tests for examining and analyzing allergenic behavior of proteins which includes the origin of a gene, sequence similarities with a known allergen, protein stability and binding mechanism of IgE epitopes. AlgPred predicted protein sequences having more than 35% sequence similarity (over 80 amino acids) with known allergens designates a protein as a potential allergen (Table 3). Based on the AlgPred result, it was observed that 29 HPs are predicted as potential allergens.

**Table 3:** Prediction of potential allergens among hypothetical proteins in *Alternaria alternata* using AlgPred tool.

S. no	Protein/amino acid residue length	Accession number	AlgPred/consesus
1.	Uncharacterized protein/379	A0A177DEM9	Allergen
2.	Uncharacterized protein/211	A0A177D895	Allergen
3.	Uncharacterized protein/549	A0A4Q4N7Y9	Allergen
4.	Uncharacterized protein/146	A0A177DEP8	Allergen
5.	Uncharacterized protein/118	A0A4Q4NGZ8	Allergen
6.	Uncharacterized protein/342	A0A4Q4NPC1	Allergen
7.	Uncharacterized protein/388	A0A177E371	Allergen
8.	Uncharacterized protein/369	A0A177D723	Allergen
9.	Uncharacterized protein/413	A0A4Q4NJR8	Allergen
10.	Uncharacterized protein/620	A0A4Q4N975	Allergen
11.	Uncharacterized protein/553	A0A177DZP1	Allergen
12.	Uncharacterized protein/187	A0A4Q4NJ87	Allergen
13.	Uncharacterized protein/608	A0A177DMN6	Allergen
14.	Uncharacterized protein/603	A0A4Q4N322	Allergen
15.	Uncharacterized protein/107	A0A4Q4N7X3	Allergen
16.	Uncharacterized protein/289	A0A177DBE8	Allergen
17.	Uncharacterized protein/418	A0A4Q4NBW2	Allergen
18.	Uncharacterized protein/612	A0A177DU49	Allergen
19.	Uncharacterized protein/187	A0A177DDT7	Allergen
20.	Uncharacterized protein/111	A0A4Q4N5B7	Allergen
21.	Uncharacterized protein/92	A0A4Q4N0I7	Allergen
22.	Uncharacterized protein/191	A0A177DIU2	Allergen
23.	Uncharacterized protein/584	A0A177DU57	Allergen
24.	Uncharacterized protein/612	A0A4Q4NRZ2	Allergen
25.	Uncharacterized protein/354	A0A4Q4NQZ6	Allergen
26.	Uncharacterized protein/493	A0A177DB16	Allergen
27.	Uncharacterized protein/183	A0A177DI22	Allergen
28.	Uncharacterized protein/107	A0A177DCS5	Allergen
29.	Uncharacterized protein/505	A0A4Q4NI20	Allergen

Bioinformatics part of guidelines 2001 has documented that a protein is potentially allergenic if it either has at least six contiguous amino acids or a minimum of 35% sequence similarity over a window of 80 amino acids shared with known allergenic proteins. The 29 protein sequences predicted as allergens by AlgPred were further analyzed with SDAP. SDAP confirms 10 protein sequences as allergens and the remaining 19 protein sequences that do not fulfill the SDAP criteria were excluded from the study (Table 4). Among the 10 protein sequences, A0A177DEP8 and A0A4Q4N5B7 showed high sequence similarity (96.25%) with the allergen Alt a 12 of *A. alternata*. Alt a 1 to Alt a 12 are the well-known allergens of *A. alternata.* Alt a 12 comprises the structure of large ribosomal protein P1, which plays a distinct role in protein synthesis^7^. A0A4Q4NGZ8 and A0A4Q4NJR8 showed a 50% sequence similarity with *Penicillium crustosum* (Pen cr 26.0101) and *Cupressus arizonica* (Cup a 3). In general, *P. crustosum* are food spoilage microorganisms and also responsible for the production of mycotoxins, in which Pen cr 26 comes under ribosomal protein P1^41^. Cupressaceae family is responsible for the relevant cause of respiratory allergy including, rhino-conjunctivitis, hay fever and asthma in sensitized individuals^42^. Cup a 3 a major allergen in this family is reactive in more than 90% of the Cupressaceae patients^43^. A0A177D895 and A0A177DB16 shown high sequence similarity (47.50%) with *Triticum aestivum* (Tri a 18) and *Gallus domesticus* (Gla d). Tri a 18 is a minor allergen for patients with bakers’ asthma^44^. A0A4Q4NI20 and A0A4Q4N975 showed 43.75% and 41.75% sequence similarity with *Musa acuminate* (Mus a 2.0101) and *Hevea brasiliensis* (Hev 5). Mus a 2 from bananas are classified under class 1 chitinase that belongs to pathogenesis-related protein (family 3), provoked positive skin prick test in 50% of banana allergic patients^45^. Hev b 5 has been identified as a major latex allergen and it is particularly observed among healthcare workers. The allergic reaction ranges from rhinitis to asthma, conjunctivitis, urticarial, anaphylactic shock, and occasionally death^46^.

**Table 4:** Screening of potential allergens in SDAP, representing its percentage identity with an allergen over a window of 80 amino acids.

S. no	Protein name	Accession number	SDAP analysis	
			FAO/WHO criteria	
			Stretches of 6 contiguous amino acid identical to an allergen	% identity with an allergen over a window of 80 a.as
1.	Uncharacterized protein	A0A177D895	Present	47.50% with Tri a 18 from a.a number 117–196
2.	Uncharacterized protein	A0A177DEP8	Present	96.25% with Alt a 12 from a.a number 1–80
3.	Uncharacterized protein	A0A4Q4NGZ8	Present	50.00% with Pen cr 26 from a.a number 14–93
4.	Uncharacterized protein	A0A4Q4NJR8	Present	50.00% with Cup a 3 from a.a number 104–183
5.	Uncharacterized protein	A0A4Q4N975	Present	41.25% with Hev b 5 from a.a number 184–263
6.	Uncharacterized protein	A0A177DU49	Present	37.50% with Act d a from a.a number 163–242
7.	Uncharacterized protein	A0A4Q4N5B7	Present	96.25% with Alt a 12 from a.a number 1–80
8.	Uncharacterized protein	A0A4Q4NRZ2	Present	37.50% with Act d a from a.a number 163–242
9.	Uncharacterized protein	A0A177DB16	Present	47.50% with Gal d 6 from a.a number 168–247
10.	Uncharacterized protein	A0A4Q4NI20	Present	43.75% with Mus a 2 from a.a number 11–90

### A0A4Q4NJR8

The functional annotations of the potential allergens are listed in Table 5. The sequence-based analysis suggests that the HP (A0A4Q4NJR8) is localized in the extracellular region and may act as a thaumatin like protein family. BLASTP search showed that the HP belongs the thaumatin-like food allergen from *Malus domestica* that is associated with IgE-mediated symptoms in apple-allergic individuals^47^. The apple protein whose amino-terminal sequence shares about 50% identity with pathogenesis-related protein-5 family members was the first thaumatin-like protein described as allergen^48^. Family and conserved domain database strongly suggest that HP belongs to the thaumatin-like protein. The thaumatin-like proteins are also involved in host defense mechanisms and a wide range of developmental processes in fungi, plants, and animals^49^. HHpred also suggests a high similarity with thaumatin I from *Thaumatococcus daniellli*. Motif search using MotifFinder suggests that the HP sequence possesses a motif that is involved in the thaumatin protein family. Usually, large type thaumatin-like protein has 16 cysteine residues at conserved positions, and this characteristic feature was also observed in our HP^50^. These residues can take part up to 8 disulfide bridges, highly conserved in the thaumatin-like proteins. String database indicates that the HP showed maximum scoring function with *Setosphaeria turcica* and the result revealed several interaction partners such as glycoside hydrolase family 2 protein, glycoside hydrolase family 12 protein, alpha glucuronidase, Arf family, and glycoside hydrolase family 30 protein. Based on these observations, we suggest that HP may function as a thaumatin protein.

**Table 5:** Sequence and structure-based functional annotation of potential allergens from the HPs of *A. alternata*.

S. no	Accession number	Sequence based functional annotation				Structure based functional annotation	
		CD-search	InterPro	Pfam	BlastP	ProFunc	DALI
1.	A0A177D895	Chitin bind 1	Chitin Binding	Chitin bind 1	Lectin-C	Crystal structure of pokeweed lectin-c	Vaccatide VH1
2.	A0A177DEP8	Ribosomal_P1 This subfamily represents the eukaryotic large ribosomal protein P1	Translational elongation	Ribosomal_60s (60s acidic ribosomal protein)	60S acidic ribosomal protein	Solution structure of human ribosomal protein p1.P2 heterodimer	60S acidic ribosomal protein p1
3.	A0A4Q4NGZ8	Ribosomal_P1 This subfamily represents the eukaryotic large ribosomal protein P1	No prediction	Ribosomal_60s (60s acidic ribosomal protein)	60S acidic ribosomal protein	Solution structure of the dimerization domain of human ribosomal protein p1/p2 heterodimer	60S acidic ribosomal protein p1
4.	A0A4Q4NJR8	Thaumatin	No prediction	Thaumatin	Thaumatin like protein	Resolution structure of the allergenic and antifungal banana fruit thaumatin-like protein at 1.7a	Thaumatin-like protein
5.	A0A4Q4N975	Glyco_hydro_1 super family	No results	Glycosyl hydrolases family 17	Beta-1,3-glucanosyltransferase	Beta-mannanase from thermomonospora fusca Mannan endo-1,4-beta-mannosidase	Glycoside hydrolase GH17 family
6.	A0A177DU49	NOB1_Zn_bind (Nin one binding (NOB1) Zn-ribbon like PIN_6 domain of ribonuclease	Cleavage involved in rRNA processing Ribosomal small subunit biogenesis	NOB1_Zn_bind (Nin one binding (NOB1) Zn-ribbon like PIN_6 domain of ribonuclease	20S-pre-rRNA-site endonuclease NOB1	Solution structure of the endonuclease nob1 from *P. horikoshii*	Pre-18S ribosomal RNA
7.	A0A4Q4N5B7	Ribosomal_P1 This subfamily represents the eukaryotic large ribosomal protein P1	Translational elongation	Ribosomal 60S (60s Acidic ribosomal protein)	60S ribosomal protein P1	TMP00 4be Solution 003: Structure of human ribosomal protein p1.P2 heterodimer	60S acidic ribosomal protein p1
8.	A0A4Q4NRZ2	NOB1_Zn_bind (Nin one binding (NOB1) Zn-ribbon like PIN_6 domain of ribonuclease	Cleavage involved in rRNA processing Ribosomal small subunit biogenesis	NOB1_Zn_bind (Nin one binding (NOB1) Zn-ribbon like PIN_6 domain of ribonuclease	20S-pre-rRNA-site endonuclease NOB1	Solution structure of the endonuclease nob1 from *P. horikoshii*	Pre-18S ribosomal RNA
9.	A0A177DB16	ChtBD1_1 (Hevein or type 1 chitin binding domain; filamentous ascomycete subfamily; Hevein or type 1	Cleavage involved in rRNA processing Ribosomal small subunit biogenesis	Chitin bind 1 (Chitin recognition protein)	Agglutinin isolectin 3	Structure of the e. Coli c-p lyase core complex Alpha-D-ribose 1-methylphosphonate 5-phosphate C-P-lyase	Agglutinin isolectin 3
10.	A0A4Q4NI20	ChtBD1_1 (Hevein or type 1 chitin binding domain; filamentous ascomycete subfamily; hevein or type 1 Chitin_bind_1 (Chitin recognition protein)	Chitin binding	Chitin_bind_1 (Chitin recognition protein) CBM_4_9 (Carbohydate binding domain)	Lectin-C	Diaminopimelate epimerase from hemophilus influenzae Diaminopimelate epimerase	Agglutinin isolectin 3

The three-dimensional structure of HP was predicted by Phyre2, which shows the sequence homology and identity of 69% and 38%, with the template (PDB ID: 2AHN). Model validation with the Ramachandran plot showed 84.9% of amino acid residues in the favored region and 0.5% of amino acid residues occupied the disallowed region. The LG-score of the HP model is − 0.835 showing that the predicted model is extremely reliable. Structure comparison and analysis revealed that the HP contains a lectin-like β barrel (Domain I), several loops (Domain II), and two beta sheets (Domain III), and all these three domains are stabilized through at least one disulfide bridge linked by up to one cysteine residues with a conserved spatial distribution throughout the protein^49^. Superimposition of the HP model with other thaumatin-like proteins showed the RMSD value of 0.660 Å (PDB ID: 3ZS3), 0.869 Å (PDB ID: 1DU5), 0.000 Å (PDB ID: 2AHN), respectively showing that HP belongs to the fold which is similar to that of 2AHN indicating a close functionality. The SuSPect tool embedded in the Phyre2 identified Cys290 amino acid residue has the highest mutational sensitivity, which has a functional/phenotypic effect in the protein. Further, Pocket-Finder analysis shows the following amino acid residues Tyr263, Asp265, Asp266, Ile268, Gln269, Arg270, Pro271, and Asn283 that plays a major role as active site residues.

DALI server shows the high structural similarity of HP with the protein function similar to thaumatin-like proteins. We found a significant match with thaumatin-like protein (Z score: 39.0), Laminaripentaose producing beta-1,3-guluase (Z score: 14.9), Beta-1,3-glucanase (Z score: 12.6), etc. The aligned residues are usually in the range of 189–412 with the RMSD in the range of 0.9 Å to 2.9 Å. We also observed a close structural similarity with the beta-1,3-glucanase enzyme. Furthermore, the ProFunc server revealed the close similarity of HP with the structure of allergenic and antifungal banana fruit thaumatin-like protein. An extensive sequence and structural analysis strongly suggest that the HP could function as a thaumatin-like protein.

### A0A177DEP8, A0A4Q4NGZ8 and A0A4Q4N5B7

The sequence (Interpro, CD search, and Pfam) based analysis strongly suggests that these HPs exist as ribosomal protein P1 and its subfamily represents the eukaryotic large ribosomal protein P1. Also, HHpred analysis showed high similarity with 60S acidic ribosomal protein P1. We found the localization of these HPs in the cytoplasm as predicted by Wolf PSORT and CELLO. The acidic ribosomal P proteins are small molecules (10–11 kDa) that form lateral stalk structures in the active site region of the large ribosomal subunit and play an important role in the elongation phase of the translation process^51^. Based on sequence homology, the ribosomal P proteins are classified into two types in mammals, yeast, and protozoans (P1 and P2), whereas, the third distinct group (P3) was observed in plants^52^. Furthermore, these HPs contain a structural motif that is found in the family of 60S acidic ribosomal protein. The functional partnership of three HPs was predicted using the STRING database which resulted in HPs (A0A177DEP8 and A0A4Q4N5B7) showing maximum scoring function with *Mycosphaerella pini* and partnership interaction with zinc-binding ribosomal protein S27e-like protein, 60S acidic ribosomal protein P0, and 0S ribosomal protein S21. Similarly, A0A4Q4NGZ8 showed maximum scoring function with *Parastagonospora nodorum* and exhibited associated functional interactions among eukaryotic ribosomal protein P1/P2 family, 60S acidic ribosomal protein P0, and universal ribosomal protein uS4 family. Based on these findings we suggest two HPs may function as 60s acidic ribosomal protein.

Three-dimensional structures of HPs (A0A177DEP8, A0A4Q4NGZ8, and A0A4Q4N5B7) were predicted using the Phyre2 server. These HPs showed high sequence similarity with the crystal structure of human ribosomal protein P1/P2 (PDB: ID-2LBF) and it was used as a template to predict the models. The predicted models were validated using the Ramachandran plot and it showed 82.1%, 84.4%, and 86.4% of amino acids were present in the favored region respectively, and none of the residues occupied the disallowed region except A0A4Q4N5B7 protein (0.7%). The model quality was validated using ProQ, which showed an LG score of − 0.835 confirming its structural quality. Likewise, structural superimposition of the predicted models with template structure showed less RMS deviation of 0.14 Å, 0.14 Å, and 0.15 Å respectively, confirming the reliability of the predicted models. DALI analysis showed similar structures that belong to 60S acidic ribosomal protein P1. Likewise, ProFunc revealed the same result as predicted by DALI. Active site prediction shows that Trp43, Leu46, Phe47, Ala50, Leu51, Lys55, Asp58, Leu59, Asn62, Val63 are the important amino acids that are essential for catalyzing A0A177DEP8 and A0A4Q4N5B7. Also, the A0A4Q4NGZ8 active site may contain Met1, Ser2, Glu9, Gln10, Ala13, Trp47, Leu50, Phe51, Ala54, Leu55, Lys58 Glu62, Val63, Leu64, Thr65, Ala66, Val67, Thr68, Ala69, and Ala70. In addition, an earlier study reports that acidic ribosomal protein P1 from *A. alternata* is considered a major allergens and plays a role in fungal allergy and autoimmune disease. Moreover, it is categorized as a rich source of mold allergens and deposited in the WHO/IUIS database^53^. The present investigation strongly suggests that these three HPs may act as 60S acidic ribosomal protein P1 and classify as allergens with the virulent property.

### A0A177D895, A0A177DB16 and A0A4Q4NI20

The sequence-based analysis including InterPro, Pfam, and CD search revealed that the HPs A0A177D895, A0A177DB16, and A0A4Q4NI20 may act as chitin recognition protein or ChtBD1_1 domain-containing protein. Also, HHpred analysis showed maximum similarity with cysteine-rich and chitin-binding proteins. Furthermore, these HPs contain a structural motif that is found in the Chitin recognition protein. The sequence-based analysis, suggests that HPs function as a chitin-binding protein. Wolf PSORT and CELLO, predict these HPs present in the extracellular region and insoluble. Chitin Binding Proteins (CBP) are involved in various biological reactions such as hydrophobic surface sensing, binding to chitin, antimicrobial activities, and increasing chitinolytic activity^54–56^. It is commonly found in the exoskeleton of arthropods, nematodes, protozoa, insects, mollusks, and fungal cell walls. Based on the chitin-binding property and amino acids similarity the carbohydrate-binding modules are classified into several families including 1, 2, 12, 14, 18, 19, and 33^57^. Chitin binding proteins mainly catalyze the chitin degradation mechanism and its action varies from fungi to other organisms. In addition, the presence of discrete domains in enzymes, and chitin-binding modules also exist as independent and non-catalytic. Such non-catalytic CBPs are mostly found in 14, 18, and 33 families^58^.

Due to the unavailability of the appropriate template, the three-dimensional structures of these HPs were predicted using the Robetta server. The quality of the structures and their accuracy were validated using the Ramachandran plot, and it showed 87.6%, 88.1%, and 87.7% of residues occupied the favored region respectively and except A0A177DB16 (0.2%) and no residues occupied the disallowed region suggested a good quality of the predicted model. LG score of − 0.835, implies the predicted models are valid with high confidence. ProFunc and DALI analysis revealed that A0A177D895 may act as chitin recognition and it is involved in a variety of biological reactions. Despite that, the other two A0A177DB16 and A0A4Q4NI20 proteins showed no significant function due to their high structural and sequence variation as compared with A0A177D895. In addition, no similar hits were obtained from ProFunc and DALI analysis. Habitually chitin, chitinases, and chitin-binding proteins produce allergenic inflammation as well as wound inducible activity. In addition, chitin-binding proteins are also classified as pathogenesis-related proteins which include prohevein and other wound-inducible proteins. Prohevein, is a cysteine-rich protein and one of the major IgE-binding allergens that affect healthcare workers in natural rubber latex. Earlier studies reported the herein protein has significant similarities with (about 71%) chitin-binding proteins which is the reason behind latex allergic patients^59,60^. Hence, the present study investigation concludes that HPs act as chitin-binding proteins and induce allergenic reactions in humans as well as cause asthmatic inflammation.

### A0A177DU49 and A0A4Q4NRZ2

HPA0A177DU49 and HPA0A4Q4NRZ2 are localized in the nuclear system. BLASTP sequence analysis suggested its activity as 20S-pre-rRNA-d site endonuclease Nin One Binding (NOB1). Furthermore, sequence-based functional prediction clearly states that HPs are the Nin One Binding (NOB1) and the virulence prediction indicates the HPs are involved in the cellular process. The 20S pre-rRNA is converted into the mature 18S rRNA in the cytoplasm due to the action of NOB1 endonuclease at site D^61^. This NOB1 contains a PilT N-terminus (PIN) domain common to many other exonucleases or endonucleases and a zinc ribbon domain. In, general, PIN domain protein has been shown to possess endonucleolytic activity^62^. Uniprot molecular function suggests that HPs possess endoribonuclease activity. String database indicates that the HP showed maximum scoring function with *Pyrenophora triticirepentis* and the result revealed several interaction partners such as bystin, pre-rRNA processing protein pno1, serine/threonine-protein kinase RIO2/RIO3, low-temperature viability protein Itv1, periodic tryptophan protein 2, U3 small nucleolar ribonucleoprotein IMP4, rRNA biogenesis protein RRP5, and GTP binding protein Bms1. MEME suite analysis suggests the presence of three significant motifs in the sequences namely 68ʹ-CHACFNIDFQMDKQFCKRC, 471ʹ-CNNDSPARYDAYAAFCKKKGAH AVGLMQD, 515ʹ-HPWEKMGDKY for both HPs. The active site region of the HPs are observed to Glu8, Ile10, Gly11, Glu12, Gly13, Thr14, Tyr15, Val18, Lys20, Ala31, Lys33, Val64, Phe80, Glu81, Phe82, Leu83, His84, Gln85, Asp86, Lys88, Lys89, His125, Asp127, Lys129, Pro130, Gln131, Asn132, Leu134, Ala144, Asp145, Ala149, Val154, Thr158, Glu162, Val163, Val164, Thr165, Trp167, Tyr168, and Leu298.

Both the HPs showed 100% sequence identity between them, therefore HP (A0A177DU49) alone was taken for structure prediction. Due to the unavailability of a reliable template, the structure of HP was predicted through an ab initio algorithm using the Rosetta server. Model validation with the Ramachandran plot showed 91.5% of amino acid residues in the favored region and 0.2% of amino acid residues occupied the disallowed region, showing high fold similarity with the template. The secondary structure prediction shows that HPs consist of numerous alpha-helices connecting through loops. The structure similarity using the DALI server shows a model that is similar to pre-18S ribosomal RNA (Z score = 29.3, RMSD = 1.5 Å), putative toxin VAPC6 (Z score = 11.7, RMSD = 4.2 Å) and Ribonuclease VAPC30 (Z score = 9.4, RMSD = 3.1 Å) etc. Moreover, structure-based function prediction using ProFunc shown that the protein may act as endonuclease nob1. Both sequence and structure-based analysis indicate that these HPs function as Nin One Binding.

### A0A4Q4N975

A0A4Q4N975 is predicted to be localized in mitochondria and extracellular as suggested by WoLF PSORT and CELLO, respectively. There is no transmembrane helix present in the sequence of HP. The motif and domain analysis suggest that the HP is a glycosyl hydrolase. The members of this glycosyl hydrolases family of enzymes have been identified in bacteria, fungi, and plants, and play key roles in different aspects of life ranging from developmental processes to host–pathogen interactions^63^. Sequence similarity search also suggests that this HP belongs to the glycoside hydrolases family 17 protein. The predicted partners for HP are endo-beta-1,3-glucanase and class III chitinase (belongs to the glycosyl hydrolase 18 families).

Due to the unavailability of any reliable template in the PDB, Rosetta was used to predict the model. The predicted model shows 86.0% of amino acid residues in the allowed region and 1.0% of residues in the disallowed region of the Ramachandran plot. Rosetta server was not able to completely predict the secondary structural elements of the HP; hence ProFunc could not able to predict the HP function. The structure similarity using the DALI server shows a model that is similar to 6FCG (Z score = 43.2, RMSD = 1.1 Å), 4WTP (Z score = 28.2, RMSD = 2.1 Å), and 3UR8 (Z score = 24.1, RMSD = 2.5 Å). Based on the sequence and structural analysis, the HP may function as glycosyl hydrolases.

## Conclusion

In the last decade, an enormous challenge has been made in characterizing the hypothetical proteins present in the genome. The functional assignments helps to understand the molecular biology at the system level and also identify potential drug targets, which can specifically act on pathogens to combat the pathogenicity. In this present study, computational analysis was performed to analyze allergic assessments of hypothetical proteins in *A. alternata*. Based on the analysis, 10 proteins were predicted as potential allergens. Furthermore, we have characterized the functions of these HPs with a high level of confidence using various bioinformatics approaches. The predicted functions of the HPs are chitin binding, ribosomal protein P1, thaumatin-like protein, glycosyl hydrolase, and Nob1 Zn binding protein. The physicochemical properties of the proteins help in the characterization of protein function, whereas subcellular localization of the proteins plays a pivotal role in differentiating the vaccine and drug targets. This study provided a basic understanding of the potential allergens and could aid in the development of novel therapeutics to counterattack *A. alternata* and other associated fungal allergic infections.

## Data Availability

The datasets used and/or analysed during the current study available from the corresponding author on reasonable request.
